# Psychosocial Risk Factors for Overuse Injuries in Competitive Athletes: A Mixed-Studies Systematic Review

**DOI:** 10.1007/s40279-021-01597-5

**Published:** 2021-12-03

**Authors:** Ulrika Tranaeus, Simon Martin, Andreas Ivarsson

**Affiliations:** 1 Department of Sport and Health Sciences, The Swedish School of Sport and Health Sciences, GIH, 114 86, Box 5626, Stockholm, Sweden; 2 Institute of Environmental Medicine, Karolinska Institutet, Stockholm, Sweden; 3 APERE, UPJV, Amiens, France; 4 School of Health and Welfare, Halmstad University, Halmstad, Sweden; 5 Department of Sport Science and Physical Education, University of Agder, Kristiansand, Norway

## Abstract

**Background:**

While the psychosocial risk factors for traumatic injuries have been comprehensively investigated, less is known about psychosocial factors predisposing athletes to overuse injuries.

**Objective:**

The aim of this review was to systematically identify studies and synthesise data that examined psychosocial risk factors for overuse injuries in athletes.

**Design:**

Systematic review.

**Data Sources:**

MEDLINE, Web of Science and PsycINFO databases, supplemented by hand searching of journals and reference lists.

**Eligibility Criteria for Selecting Studies:**

Quantitative and qualitative studies involving competitive athletes, published prior to July 2021, and reporting the relationship between psychosocial variables and overuse injury as an outcome were reviewed. This was limited to academic peer-reviewed journals in Swedish, English, German, Spanish and French. An assessment of the risk of bias was performed using modified versions of the RoBANS and SBU Quality Assessment Scale for Qualitative Studies.

**Results:**

Nine quantitative and five qualitative studies evaluating 1061 athletes and 27 psychosocial factors were included for review. Intra-personal factors, inter-personal factors and sociocultural factors were found to be related to the risk of overuse injury when synthesised and reported according to a narrative synthesis approach. Importantly, these psychosocial factors, and the potential mechanisms describing how they might contribute to overuse injury development, appeared to be different compared with those already known for traumatic injuries.

**Conclusions:**

There is preliminary evidence that overuse injuries are likely to partially result from complex interactions between psychosocial factors. Coaches and supporting staff are encouraged to acknowledge the similarities and differences between traumatic and overuse injury aetiology.

**Supplementary Information:**

The online version contains supplementary material available at 10.1007/s40279-021-01597-5.

## Key Points

**Table:** 

The findings in this review identified potential psychosocial risk factors for overuse injuries.
The 27 identified factors were categorised into intra-personal factors, inter-personal factors and sociocultural factors. Stress was identified as one of the risk factors, which is similar to studies in traumatic injuries.
Psychosocial risk factors for overuse injuries are an underexplored area. Prospective studies with repeated measures are needed in future studies, as well as an agreement over the definition and operationalisation of these types of injuries.

## Introduction

Overuse injuries are highly prevalent in sports with repetitive movement such as athletics [1], tennis [2], volleyball, handball, cycling, floorball [3] and swimming [4]. These injuries are problematic because they are related to negative consequences such as poorer performance [5], high cost for rehabilitation [6] and retirement from sport [7]. Nearly one out of two former professional soccer players retired from English professional football because of injury, of which 58% were overuse injuries [8]. The pathology of overuse injuries is different from traumatic injuries and the aetiology needs to be investigated before preventive strategies can be evaluated.

In a comprehensive model for injury causation, internal and external risk factors have been discussed [9]. The authors concluded that the inciting event can be distant from the occurrence of an injury. This is because all injuries do not occur at a single event even though the pain can have an acute onset, for example overuse injuries. Overuse injuries develop most often because of repetitive loading of the musculoskeletal system without adequate rest that allows the structures to adapt to the training load and may occur suddenly without identified events [10–12], while traumatic injuries occur at an identified specific event with or without contact with another person or object, for example ankle sprain [13, 14]. An imbalance between training load and recovery was therefore described as a key factor for explaining how overuse injuries may occur [15–17]. Risk factors for overuse injuries have mainly been described in terms of training load, [16] cyclic chain of shifting circumstances [18], performance level and previous injury [19]. Because of the gradual onset of an overuse injury, a multifactorial explanation including bio-psychosocial factors is more evident [20], for example psychosocial stress reduces the muscle recovery after resistance training [21]. Within the Biopsychosocial Model of Stress, Athletic Injury and Health (BMSAIH) [20], different pathways between psychosocial stress and athletic injuries are suggested. More specifically, psychophysiological stressors (e.g. negative life-event stress, physical training) are suggested to influence the autonomic nervous system, which in turn influences recovery and behavioural mechanisms (e.g. decreased self-care and sleep quality) [20]. The changes in recovery and behavioural mechanisms may, in the next step, increase the risk for overuse injuries. Another multifactorial model discussing the aetiology of overuse injuries is the overtraining risks and outcomes model [22]. Within this model, the interactions between psychosocial, intra-personal, inter-personal and situational factors are suggested to influence the risk of imbalance between stress and recovery. Factors discussed in this model include super-motivation, pushing through injuries, relationships and behaviours of others related to injuries [22].

Psychosocial risk factors for traumatic injuries and overuse injuries in sport have previously been investigated. For example, athletes who experienced changes and a high level of stressful events were at a greater risk for sustaining a traumatic injury while no relation to overuse injuries was found [23, 24]. An empirical risk factor model [25, 26] that included psychosocial factors was suggested to influence an athlete’s stress response. An evaluation of the model showed that stress and a high stress response were related to an increased risk for injuries, but most of the included studies used time loss as the injury definition and very few studies separated overuse injuries and traumatic injuries [27]. Overuse injuries seldom result in time loss [11, 28] and are not adequately captured in these types of studies and evaluations.

Because of the prevalence of overuse injuries, it is of interest to explore psychosocial risk factors for overuse injuries. Particularly, it is important to understand and explain why and how overuse injuries occur in line with the studies regarding psychosocial risk factors for traumatic injuries. With that knowledge, effective prevention programmes can be developed, implemented and evaluated. The aim of this study was to systematically review studies examining psychosocial risk factors for overuse injuries in competitive athletes.

## Methods

For conducting and reporting this systematic review, we followed the Preferred Reporting Items for Systematic Reviews and Meta-analyses (PRISMA) statement [29], see Appendices 1 and 2 of the Electronic Supplementary Material (ESM). The study protocol was prospectively registered (PROSPERO ID: CRD42019123580).

### Definitions and Outcome

Overuse injury was defined as an injury occurring without an identified inciting event [10, 30]. The risk factors were psychosocial factors that were identified to influence the risk for the occurrence of overuse injury.

### Literature Search Strategy

Medline (Ovid), Web of Science Core Collection and PsycINFO (Ovid) were searched from inception to July 2021. Hand searching of journals and reference checking were also performed by the authors.

The following keywords were used together with other related words, and with appropriate truncations and Boolean combinations of words and operators: “overuse injury” AND “sport” AND “psychology” AND “risk factor” limited to academic peer-reviewed journals in Swedish, English, German, Spanish and French. A complete search was performed by librarians at Karolinska Institutet, Sweden after several test searches. See Appendix 3 of the ESM for a full documentation.

### Eligibility Criteria/Selection Criteria

Studies reporting psychological or psychosocial risk factors for overuse injuries in athletes published in academic peer-reviewed journals in the above-mentioned languages until July 2021 were eligible for quality assessment. Eligible studies had to include competitive athletes as a population*.* Studies where the outcome was not clearly stated, or where overuse injuries were pooled with other injuries (e.g. traumatic or chronic injuries), were excluded.

Published papers without empirical data, not presenting results about overuse injuries or not assessing psychosocial factors, were excluded as well as duplicates. Articles assessing psychosocial factors as an outcome after overuse injury were also excluded.

### Risk of Bias Assessment

The risk of bias was evaluated using a modified Risk of Bias Assessment tool for Non-randomized Studies, RoBANS [31], i.e. items specific to overuse injuries and “psychological factors” were included (see Appendix 4A of the ESM for a full description). RoBANS is a six-item tool for assessing selection bias of participants and confounding variables, misclassifications bias, attrition bias, reporting bias and analysis in non-randomised studies. For each item, a low, unclear or high risk of bias was evaluated according to the specified criteria [31]. For assessment of qualitative studies, we used the “Quality assessment scale for qualitative studies” by the Swedish Agency for Health Technology Assessment and Assessment of Social Services [32] (see Appendix 4B of the ESM for a full description). These two assessment tools are frequently used and were chosen with the assumption that most of the included articles would be non-randomised and qualitative studies.

### Data Extraction and Synthesis

Two of the authors (UT, SM) independently screened and extracted the articles by title and abstract using an online screening tool, Rayyan [33]. The remaining articles were assessed in full text and the quality and bias were evaluated by the two first authors using the assessment tools. Any discrepancies were resolved by consensus or by involving the third author (AI).

A decision not to perform a meta-analysis was taken after pilot searches revealed substantial methodological and clinical heterogeneity between studies. Instead, data from both quantitative and qualitative studies were synthesised and reported according to the narrative synthesis approach, commonly referred to as the best approach to “tell the story” of the findings from a wide range of research designs [34] and categorised into three areas: intra-personal and inter-personal and sociocultural factors. We reported effect sizes (specified in-text) when available in quantitative studies.

## Results

### Literature Identification

**Fig. 1:**
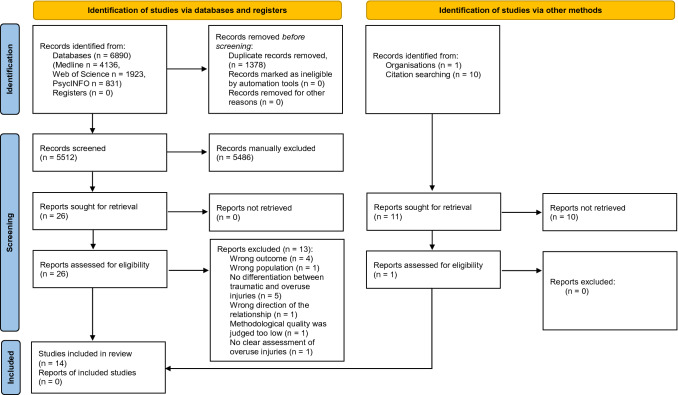
Preferred reporting items for systematic reviews and meta-analyses (PRISMA) flow diagram

Systematic database searching yielded a total of 6890 records, and a further 11 studies were identified through other sources (e.g. citation searching). Following deletion of duplicates, a first screening based on title and abstract resulted in selection of 26 eligible articles. Full-text articles were subsequently obtained and assessed against eligibility criteria (see Appendix 5 of the ESM for full references and reasons for exclusion), leaving 14 articles included for a full review and synthesis. Nine studies reported quantitative associations between psychosocial factors and overuse injury, while five studies reported qualitative findings. For an overview of the screening process, see Fig. 1.

### Study Characteristics

The 14 studies included data from 1061 athletes with a mean age of 25.9 years, of whom 589 (56%) were female and 472 (44%) were male. The competitive level ranged from regional to international. The 14 studies covered a wide variety of individual and team sports. See Table 1 for a detailed description of the study and sample characteristics.

**Table 1:** Study and sample characteristics

Study	Design (duration)	Definition and measure of overuse injuries	Population	Sample size (having reported at least one overuse injury)	Sex/gender (M:F)	Mean age (years)	Psychological factors investigated	Measures
Quantitative studies								
Berengüí et al. [35]	Retrospective study	Ad-hoc self-reported information about overuse injuries. A group of sport medicine experts used the Orchard Sports Injury Classification System to confirm overuse injury diagnosis	Spanish athletes competing at an international level in various sports	38 (not reported)	27:11	21.4	Personality traits (e.g. vigilance, tension, reasoning)	Sixteen Personality Factor Questionnaire, 5th Edition (16PF-5)
Christensen and Ogles [36]	Prospective cohort study (9 months)	Overuse injuries were self-reported retrospectively (follow-up survey) using a modified version of the Injury Checklist. An overuse injury was detected when participants reported a running-related cause of injury such as intensity of training, overuse, or repetitive use	Marathon runners of various levels in the USA	162 (42)	81:81	40.8	Attentional focus; motivation/ competitiveness; exercise dependency	Attentional Focus Questionnaire (AFQ); Motivations of Marathoners Scales (MOMS); Obligatory Exercise Questionnaire (OEQ)
Ekenman et al. [37]	Retrospective study	Tibial stress fracture diagnosed with a confirmatory x-ray, bone scintigram or MRI	Swedish long-distance runners of various levels	34 (17)	16:18	37.2	Type- A behaviour; locus of control; exercise dependency; motivation/ competitiveness; gender typing	Heart and Lifestyle Type A Measure (HALTAM); Jenkins Activity Survey for Sweden (JAS-S); Rotter Internal–External Control Scale (RIECS); Commitment to Exercise Scale (CtES); Reason for Exercise Inventory (REI); Competitiveness scale; Participation Motivation Questionnaire (PMQ); Motivation for Physical Activities Measure (MPAM); Bem Sex Role Inventory (BSRI)
Martin et al. [38]	Prospective cohort study (10 weeks)	Weekly self-reported overuse injuries using the Oslo Sports Trauma Research Centre (OSTRC) Overuse Injury Questionnaire and based on a functional definition (including but not restricted to time-loss injuries). Post-hoc confirmation of the overuse diagnosis using data recorded by the head coaches on traumatic injuries (discrimination procedure) and individual phone interviews	Regional to international- level athletes competing in various individual or team sports in France or Sweden	149 (135)	105:44	27.9	Athletic identity; perfectionism; perceived stress from sport and life; coach-athlete relationship	Athletic Identity Measurement Scale (AIMS); Brief Frost-Multidimensional Perfectionism Scale (F-MPS); Life Event Survey for Collegiate Athletes (LESCA); Coach–Athlete Relationship Questionnaire (CART-Q)
Pensgaard et al. [39]	Prospective cohort study (12 months)	Self-reported, time -loss (at least 1one day) overuse injuries. Diagnosis confirmed by a physiotherapist using a standardized interview based on the Orchard Sports Injury Classification System	Elite female football players competing in the Norwegian elite league	193 (55)	0:193	21.6	Inter-personal stressors; perceived motivational climate	Life Event Survey for Collegiate Athletes (LESCA); Perceived Motivational Climate in Sport Questionnaire (PMCSQ)
Timpka et al. [40]	Prospective cohort study (52 weeks)	Weekly self-reported overuse injuries using the following definition: “a condition to which no identifiable single external transfer of energy could be associated but that caused changes in the mode, duration, intensity or frequency of normal training/competition”	Swedish elite track and field athletes, ranked in the national top 10 in youth or adult age groups	266 (184)	118:148	24	Body consciousness and hyperactivity; coping skills; perceived motivational climate; exercise dependency	Body Consciousness Scale (BCS); items from the hyperactivity definition in the Diagnostic and Statistical Manual of Mental Disorders, Fourth Edition (DSM-IV); Brief Cope (BC); Perceived Motivational Climate in Sport Questionnaire (PMCSQ); Commitment to Exercise Scale (CtES)
van der Does et al. [41]	Prospective cohort study (41 weeks)	Overuse medical attention injuries were recorded by the team’s physical therapist using the following definition: “an injury caused by repeated microtrauma without a single, identifiable event responsible for the injury”	Athletes playing in regional or national-level indoor team sports in the Netherlands who were enrolled in a larger study known as the Groningen Monitoring Athletic Performance Study (Groningen MAPS)	86 (not reported)	58:28	21.9	Perceived stress from sport and life	RESTQ-Sport
Van der Sluis et al. [42]	Prospective cohort study (32 weeks)	Weekly self-reported overuse injuries using the Oslo Sports Trauma Research Centre (OSTRC) Overuse Injury Questionnaire and based on a functional definition (including but not restricted to time-loss injuries). Diagnosis continuously confirmed by physicians	Young elite tennis players participating in the national high-performance program of the Royal Dutch Lawn Tennis Association (The Netherlands)	73 (54)	45:28	12.4	Risk- taking	Iowa Gambling Task (IGT)
Van der Sluis et al. [43]	Prospective cohort study (32 weeks)	Weekly self-reported overuse injuries using the Oslo Sports Trauma Research Centre (OSTRC) Overuse Injury Questionnaire and based on a functional definition (including but not restricted to time-loss injuries). Diagnosis continuously confirmed by physicians	Young elite tennis players participating in the national high-performance programme of the Royal Dutch Lawn Tennis Association (The Netherlands)	73 (54)	45:28	12.4	Meta-cognitive skills of self-regulation	Self‐Regulation of Learning Self‐Report Scale (SRL‐SRS)
Qualitative studies								
Cavallerio [44]	Ethnographic study (12 months)	Not reported. Overuse injury was defined as a dynamic and complex process more than as an outcome	Rhythmic gymnasts training in one elite club in Italy	16 (not reported)	0:16	13.6	How sport culture impacts overuse injuries	
Jelvegård et al. [45]	Semi-structured interviews	Not reported. However, a difference was made between illnesses, sudden- onset injuries and gradual- onset (overuse) injuries	Swedish middle-distance and long-distance runners from the national top 15 list	14 (not reported)	8:6	Range = 21–36	Athletes’ cognitive interpretations of body perceptions and subsequent behavioural responses preceding overuse injury	
Russell and Wiese-Bjornstal [46]	Narrative inquiry (semi-structured interviews)	Self-reported microtrauma, defined as an injury occurring due to the accrual of repeated small forces over a period of time as a result of long-distance running training, sustained within the 18 months prior to recruitment	Novice to experienced American long-distance runners (5- km races and more)	10 (10)	2:8	25.1	The chronology of psychosocial experiences and responses associated with overuse injuries	
Tranaeus et al. [47]	Semi-structured interviews	Overuse injuries, defined as the result of sub-maximal repetitive mechanical load in the affected tissue without trauma, diagnosed by the team medical staff	Elite floorball players competing in the Swedish premier League	11 (11)	9:2	25	Athletes’ beliefs regarding the psychological risk factors for overuse injuries	
van Wilgen and Verhagen [48]	Semi-structured interviews	Prior to the interview, athletes having recently experienced an overuse injury were asked for the onset of the injury to ensure these were not being confused with traumatic injuries	Non-professional, national- level athletes competing in various sports in the Netherlands	9 (9)	3:6	21.3	Athletes and coaches’ beliefs regarding the psychological risk factors for overuse injuries	

M:*F* female, *M* male, *MRI* magnetic resonance imaging:female

### Measures

In total, 27 psychosocial factors were identified. Of those, 17 factors were highlighted in the quantitative studies using 26 different measures (see Table 1). Two studies reported on the development of new scales to measure psychosocial factors (competitiveness and hyperactivity) [37, 40]. All studies reported information about psychometric properties of the psychosocial measures used, for which internal consistency ranged from acceptable (0.7 ≤ *α* < 0.8) to excellent (0.9 ≤ *α*). All studies used single timepoint measures of psychosocial variables (e.g. as baseline measures in prospective studies), except for the study of van der Does et al. [41] in which perceived stress and recovery were measured every 3 weeks.

Methods for measuring overuse injuries also varied between studies (see Table 1), mostly because of the different definitions and diagnosis methods used. Ten studies used self-reported measures [35, 36, 38–40, 42, 43, 45, 46, 48], including a subsequent diagnosis confirmation by medical professionals for six of them [35, 38, 39, 42, 43, 48], whereas three studies used medical attention as a condition for diagnosis of an overuse injury [37, 41, 47]. One study referred to the time-loss definition [39], while four studies clearly mentioned the use of a functional definition [38, 40, 42, 43]. The two studies of Van der Sluis et al. were conducted on the same sample [42, 43].

### Quantitative Studies

#### Psychosocial Factors

The 17 psychosocial factors identified in the nine quantitative studies were clustered into three different categories: intra-personal factors, inter-personal factors and sociocultural factors.

##### Intra-Personal Factors

Fourteen intra-personal factors were examined in the included quantitative studies: motivation/competitiveness, exercise dependency, athletic identity, perceived stress from sport and life, type A behaviour, perfectionism, risk taking, coping skills, personality traits, attentional focus, locus of control, gender typing, metacognitive skills of self-regulation, and body consciousness and hyperactivity.

Athletes having reported an overuse injury scored higher in competitive and goal-oriented motivation in comparison to their counterparts [36]. However, the level of competitiveness could not be used to discriminate between athletes with and without overuse injury [37]. Female athletes with an overuse injury scored as high, or higher, than athletes without overuse injury on a number of subscales measuring motivation for exercise, namely: weight management, physical health, stress-mood, skill development, fun-enjoyment, socialising and muscle improvement [37]. However, none of these differences was statistically significant, and these differences were not observed in men. Exercise dependency was found to be associated with overuse injury risk in marathon runners [36], and in female long-distance runners [37], but not in elite track and field athletes [40]. The combination of competitive motivation, goal-oriented motivation and exercise dependency increased the risk for overuse injuries [36]. Athletes categorised in the group with the highest risk for overuse injuries were characterised by a higher level of athletic identity in comparison to athletes in the other two groups, who reported fewer overuse injuries [38].

No significant differences were found between athletes who sustained overuse injuries and uninjured athletes regarding absolute perceived stress and recovery [41]. However, a decrease in perceived personal accomplishment in sport over 3 weeks of training increased the risk of sustaining an overuse injury during the following period (OR = 0.59) [41]. One study showed that perceived negative life event stress was the main variable allowing discrimination between athletes in a psychosocial risk profile for overuse injuries (who presented with elevated stress values) and athletes in the other profiles who were less prone to negative life event stress and who sustained fewer overuse injuries [38]. Female athletes with a previous overuse injury were found to score significantly higher than the other women for overall type A behaviour and for the sub-dimension time pressure [37]. Athletes in a psychosocial risk profile for overuse injuries were characterised by higher values for perfectionistic concerns than athletes in the other profiles, whereas perfectionistic strivings did not contribute to discriminating at-risk athletes for overuse injury [38]. In young male athletes, risk taking explained 15% of the variance in time-loss overuse injuries and 13% of the variance in overuse severity scores [42]. The maladaptive coping behaviour of self-blame was found to be associated with an increased risk of overuse injury in athletes [40].

Athletes who reported an overuse injury scored higher values for vigilance (Cohen’s* d* = 1.003), privateness (*d* = 0.758) and self-reliance (*d* = 0.943) and lower values for dominance (*d* = 0.716), rule consciousness (*d* = 0.944) and overall self-control (*d* = 1.178) in comparison to injury-free athletes [35]. No differences were found for the other ten personality traits that were measured in this study [35]. Regarding their attentional focus, athletes who sustained an overuse injury had a significantly higher preference for association with internal physiological sensations while training (as opposed to dissociation) than athletes without overuse injury [36], but not when attentional focus was considered as a potential mediator between motivational variables and injury [36]. Locus of control did not discriminate between athletes with and without overuse injury [37]. No statistically significant differences were found between athletes with a previous overuse injury and injury-free athletes for any of the attributes (i.e. instrumentality, expressiveness and social desirability) that are used to define individuals’ gender typing [37]. In another study, a lack of self-monitoring skills was found to be associated with a higher category of time-loss overuse injuries (odds ratio = 4.555). This was particularly the case in girls (odds ratio = 10.757), but not in boys [43]. In addition, the reflection score significantly predicted overuse injury severity score along with exposure time (*R*^2^ = 0.201) [43]. The other two meta-cognitive skills, planning and evaluation, were not related to the risk of overuse injury [43]. No differences were found in overuse injury risk among athletes regarding body consciousness and hyperactivity used as a combined variable [40].

##### Inter-Personal Factors

Two inter-personal factors were identified in the quantitative studies: coach-athlete relationship and inter-personal stressors. Athletes categorised into the psychosocial risk profile for overuse injuries reported having a relatively poor relationship with their coach, in comparison with the other profiles [38]. Athletes reporting their coach as a source of stress were found to be at greater risk of sustaining an overuse injury (odds ratio = 1.21) [39]. In this study, none of the other inter-personal stressors investigated (teammates and friends) was associated with overuse injury risk [39].

##### Sociocultural Factors

A single sociocultural factor was investigated in the quantitative studies: perceived motivational climate. This factor assesses the athletes’ perceptions of the motivational climate within their teams using an ego-oriented climate and a task-oriented climate. None of the two variables of perceived motivational climate, ego-oriented climate and task-oriented climate, was found to be associated with the risk of overuse injury [39, 40].

#### Assessment of Risk of Bias

The results of the assessment of risk of bias for quantitative studies are presented in Table 2. One of the nine studies was rated as having a low risk of bias [37], four had an unclear risk of bias for some items, [38–40, 42], whereas the other four studies were allocated a high risk of bias for at least one item [35, 36, 41, 43].

**Table 2:**
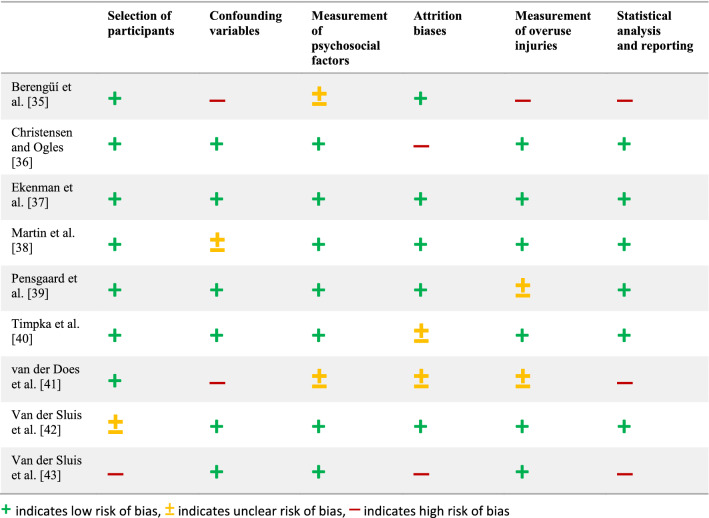
Assessment of risk of bias for quantitative studies (modified RoBANS)

### Qualitative Studies

#### Psychosocial Factors

Sixteen psychosocial factors were identified in the five qualitative studies and were classified in the same three categories as we used for the classification of variables from the quantitative studies: intra-personal factors, inter-personal factors and sociocultural factors.

##### Intra-Personal Factors

Nine intra-personal factors were examined in the identified qualitative studies: motivation/competitiveness, athletic identity, passion/dedication, excessive training, neglecting warnings signals and long-term consequences, acceptance of pain/decreased function, perceived stress from sport and life, previous injuries and coping skills.

Having set a clear goal corresponding to the date of a competition was described as predisposing athletes to overuse injuries [46]. A strong athletic identity characterised athletes having sustained an overuse injury in one qualitative study [47]. Passion and dedication to their sport were also reported as risk factors predisposing athletes to overuse injuries in three different studies [44, 47, 48]. Excessive training [47, 48] as well as training despite being mentally and/or physically tired [47], were described as behaviours increasing the risk for overuse injuries. A range of cognitive interpretations that followed the perception of the gradual overuse symptoms were described as psychosocial mechanisms resulting in more severe or prolonged overuse injury episodes [45, 47, 48]. In the early stages of overuse injuries, athletes expressed that they ignored the bodily warning signals and neglected the possible negative long-term consequences of training despite these symptoms [47, 48]. In the following stages, athletes’ thought patterns included ‘magical thinking’, meaning that the problem will resolve by itself without having to change their training behaviours [45]. In the late stages of overuse injuries, athletes were found to accept the pain and decreased function associated with the injury and to continue training and competing, unless the pain had increased to an intolerable level or if strong recommendations were received from medical professionals or coaches to adapt their training [45, 47]. The common thread in these cognitive interpretations was that they all allowed the athletes to avoid resting.

Sport-specific stressors (e.g. insecure position in the team) and non-sport stressors (e.g. stress from work or school) were also reported as risk factors for overuse injuries [47, 48]. In addition, a lack of recovery emerged as a common risk factor in one study [47]. Putting too much pressure on oneself was a personal stressor identified as a risk factor in two qualitative studies [47, 48]. Having sustained previous injuries was also described by athletes as a risk factor for subsequent overuse injuries, in the sense that they were aware of what recognising themselves as injured again would mean in terms of absence from training, low self-efficacy and negative emotions associated with the rehabilitation period [47]. These athletes therefore preferred not to consider their overuse symptoms as reflecting an injury, a reasoning that is likely to result in a more serious overuse injury [47]. Athletes having sustained an overuse injury reported a lack of adaptive skills to cope with pressure and fear [47, 48], and to handle physical complaints [47].

##### Inter-Personal Factors

Five inter-personal factors were identified in the qualitative studies: coach-athlete relationship, communication, internal rivalry, inter-personal stressors and social support. A poor coach-athlete relationship was perceived as a contributing factor to overuse injuries in one study [48]. Additionally, good relationships may also be linked to an increased risk of overuse injury as participants reported their sense of duty towards a coach (or team) as a potential risk factor [48]. Poor communication between athletes and their coaches, or a misalignment between different coaches, was suggested to increase the risk of overuse injury by creating misperceptions and by encouraging athletes not to disclose their early symptoms [44, 47, 48]. Internal rivalry was also expressed as contributing to the onset of overuse injuries [47, 48]. Inter-personal stressors involving other individuals (i.e. the club’s president, coaches, teammates and the audience) were also reported as factors that contributed to the onset of overuse injuries [44, 47]. The overall lack of social support from family, friends and teammates, as well as the specific lack of social support from coaches and medical staff when facing an overuse injury were also reported by these athletes [47].

##### Sociocultural Factors

Two sociocultural factors were identified in the qualitative studies: pain normalisation and the belief that overuse injuries are less important than traumatic injuries. Pain normalisation was described as the core feature of a ‘culture of risk’, which is associated with a low acceptance of complaining [44, 47]. These athletes were described as ensuring their cultural embodiment by showing their adherence to the social values of their club (e.g. sporting success, striving for perfection) through ‘mentally tough’ attitudes and behaviours. This meant accepting pain as an integral part of sport and continuing to train and compete despite experiencing pain, which ultimately resulted in overuse injuries [44]. The social norm that overuse injuries are less important than traumatic injuries and do not necessitate serious consideration was also apparent in three qualitative studies conducted in different contexts [44, 47, 48].

#### Assessment of Risk of Bias

The results of the risk of bias assessment for qualitative studies are presented in Table 3. One study was rated as having a low risk of bias [47]. Three of the five studies were rated as having an unclear risk of bias for two items [44–46], whereas the remaining study was allocated a high risk of bias for one item [48].

**Table 3:**
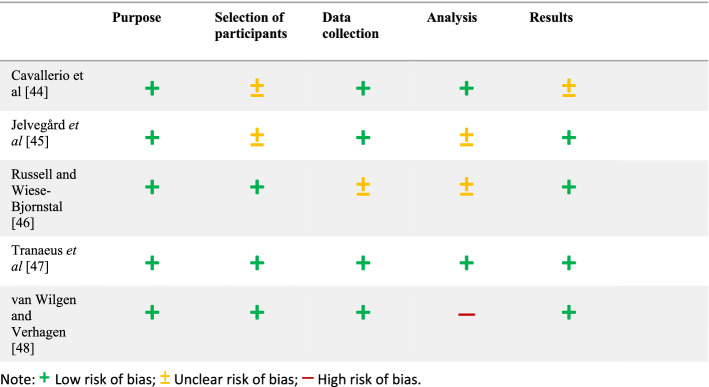
Assessment of risk of bias for qualitative studies (modified SBU Quality Assessment Scale for Qualitative Studies) [32]

### Meta-Synthesis: Summary of the Findings

A meta-synthesis table summarising the findings of both quantitative and qualitative studies and providing an overview of the certainty of evidence for each factor is presented in Table 4.

**Table 4:** Meta-synthesis: summary of the findings

Psychosocial factors	Findings	References	
		Quantitative studies	Qualitative studies
Intra-personal factors			
Motivation/competitiveness	Possible effect of goal-oriented motivation; conflicting results for other types of motivation	[36, 37]	[46]
Exercise dependency	Conflicting results	[36, 37, 40]	
Athletic identity	Possible effect of athletic identity	[38]	[47]
Perceived stress from sport and life	Possible effect of perceived stress; conflicting results for stress and recovery imbalance	[38, 41]	[47, 48]
Type-A behaviour	Possible effect of overall Type A behaviour and the sub-dimension time pressure	[37]	
Perfectionism	Possible effect of perfectionistic concerns; no effect of perfectionistic strivings	[38]	
Risk- taking	Possible effect of risk-taking in male athletes; no effect in female athletes	[42]	
Coping skills	Possible effect of using the maladaptive coping behaviour of self-blame and of a lack of coping skills	[40]	[47, 48]
Personality traits	Possible effect of vigilance, privateness and self-reliance; possible effect of lack of dominance, rule- consciousness, and overall self-control; no effect for the other personality traits	[35]	
Attentional focus	Conflicting results	[36]	
Locus of control	No effect of locus of control	[37]	
Gender typing	No effect of gender typing	[37]	
Metacognitive skills of self-regulation	Possible effect of lack of self-monitoring skills, especially in girls; conflicting results for reflection; no effect of planning and evaluation	[43]	
Body consciousness and hyperactivity	No effect of body consciousness and hyperactivity	[40]	
Passion/dedication	Possible effect of passion/dedication		[44, 47, 48]
Excessive training	Possible effect of excessive training/training despite fatigue		[47, 48]
Neglecting warnings signals and long-term consequences	Possible effect of neglecting warnings signals and long-term consequences		[45, 47, 48]
Acceptance of pain/decreased function	Possible effect of acceptance of pain/decreased function		[45, 47]
Previous injuries	Possible effect of previous injuries		[47]
Inter-personal factors			
Coach-athlete relationship	Possible effect of a poor coach-athlete relationship or of a sense of duty towards a coach (or team)	[38]	[48]
Inter-personal stressors	Possible effect of perceived stress from the coach, the club’s president, and the audience; conflicting results for perceived stress from teammates/friends	[39]	[44, 47]
Communication	Possible effect of a bad communication between athletes and coaches or between different coaches		[44, 47, 48]
Internal rivalry	Possible effect of internal rivalry		[47, 48]
Social support	Possible effect of perceived lack of social support		[47]
Sociocultural factors			
Perceived motivational climate	No effect of perceived motivational climate	[39, 40]	
Pain normaliszation	Possible effect of pain normaliszation		[44, 47]
The belief that overuse injuries are less important than traumatic injuries	Possible effect of the belief that overuse injuries are less important than traumatic injuries		[44, 47, 48]

Meta-synthesis of the main findings from quantitative and qualitative studies (a possible effect indicates a higher risk of overuse injury). Total: 14 studies, 1061 competitive athletes

## Discussion

The aim of this review was to systematically identify psychosocial risk factors for overuse injuries in competitive athletes. Overall, we identified nine quantitative and five qualitative studies with a focus on 27 psychosocial risk factors for overuse injury in various sports and athletic levels. Based on the results from these studies, we suggest that a number of intra-personal, inter-personal and sociocultural factors might influence the risk of overuse injuries and should, therefore, be considered in sports burdened by overuse injuries. However, the certainty of evidence around the psychosocial risk factors for overuse injuries remains small in comparison to the evidence for traumatic injuries. Consequently, the preliminary findings presented in this review may provide grounds for further exploration of these potential risk factors.

Importantly, the relatively high risk of bias identified in a majority of the included studies should be considered when interpreting and using the present findings. There are several aspects that are important to highlight in relation to this issue. First, regarding the certainty of evidence of the psychosocial factors identified, it should be noted that some variables were only investigated in single studies (see Table 4). Second, an important aspect in relation to the strength of evidence is the heterogeneity of the overuse injury definitions and recording methods used in the included studies. However, most of them (10 out of 14) used the recommended self-reported method [65], and only one study referred to the time-loss definition that should be avoided [65]. Third, the athletic level may be a potential confounding factor when examining the risk of overuse injury. While studies dealing with recreational-level athletes were excluded, this review covered a wide range of competitive levels (from regional to international), which should be considered when interpreting the present findings. However, the potential risk factors identified in our study (e.g. passion, athletic identity) suggest that the level of investment in the sport of athletes might be more important than their absolute level of performance in relation to overuse injuries. Consequently, attention should be directed towards athletes of all levels who may be equally susceptible to overuse injuries. Moreover, some of the factors identified such as sociocultural factors (e.g. pain normalisation) are likely to be sport dependent.

Despite the uncertainty of the evidence, the results of the included studies indicate that psychosocial factors might increase the risk of overuse injuries. More specifically, psychosocial stress, whether involving intra-personal or inter-personal stressors, appeared to be one of the most prominent factors identified in the current review [38, 39, 41, 44, 47, 48]. Athletes exposed to psychosocial stress may be more susceptible to overuse injuries through the synergetic effects of psychosocial and physical stress [20, 49]. This hypothesis is supported by previous research indicating that athletes’ adaptation to intense training is impaired by psychosocial stress [21, 50] and concordant with the primary tenet of the biopsychosocial model of stress and athletic injury and health [20]. More specifically, emotional, behavioural and physiological factors should be considered as potential mechanisms mediating the relationship between psychosocial stress and overuse injuries [20]. Indeed, overuse injuries are considered to be a response at the cellular level of repetitive overload at the systemic level [51], and chronic exposure to psychosocial stressors might contribute to this systemic overload through immune and hormonal patterns [20, 49]. Cognitive features are also known to exacerbate or prolong emotional reactivity to a stressor and the concomitant physiological response [20]. Subsequently, individuals may gradually accommodate to their overuse injury because the initially prominent affective reaction becomes weaker and receives decreased attention [45, 56], and the affected individuals therefore progressively accept their impaired function [45, 47].

In line with a complex approach to sport injury [52], the other intra-personal factors identified in this review, such as perfectionism or coping skills, are likely to interact with the identified inter-personal (e.g. communication) and sociocultural (e.g. pain normalisation) risk factors to influence the risk of overuse injury. For example, previous findings indicate that individuals with elevated perfectionistic concerns are more likely to experience chronic psychosocial stress [54, 55], thus leading to a higher risk of sustaining athletic injuries [53], which is consistent with our results. Athletes may also cope differently when experiencing physical complaints depending on certain dimensions of perfectionism [57], which may influence the development of an overuse injury. Furthermore, athletes presenting with a high athletic identity or goal-oriented motivation were suggested to be less flexible in their training because the frustration associated with having to rest may overshadow the negative emotions associated with the first signs of a developing overuse injury [37]. The inter-personal factors identified in the current review may reinforce this pattern. Poor communication and/or relationships between athletes and coaches may, for example, encourage athletes to under-report pain and early symptoms to avoid a conflict [39, 44]. The current review also found sociocultural risk factors for overuse injuries such as displaying ‘mentally tough’ attitudes and behaviours [44], which is congruent with theoretical models such as the biopsychosocial sport injury risk profile [58] and contributes to explaining why athletes with overuse injuries often delay the decision to rest and seek medical attention despite substantial impairment of performance and training capacities [11, 59].

### Practical Implications

With respect to practical recommendations, as psychosocial stress may act in synergy with physiological stress to increase the risk of overuse injuries [20, 49], coaches and clinicians should consider using broad subjective measures aiming to monitor the athletes’ response to both training and non-training (i.e. psychosocial) stressors [60]. This could be done, for example, on a daily or weekly basis using self-reported measures. Examples of such tools include the Recovery Stress Questionnaire for Athletes (RESTQ-S) [61] and the Multi-Component Training Distress Scale (MTDS) [62]. Importantly, as psychosocial stress seems of be a common antecedent of traumatic [27] and overuse injuries, these monitoring tools, as well as psychological interventions targeting stress responses (e.g. mindfulness-based programmes [63]) might be implemented to prevent both types of injuries. We encourage coaches for which these measures might not be affordable to introduce communication routines with their athletes on a daily basis, for example by taking a few minutes at the beginning of every new training session to discuss their level of recovery and experience of potential stressors, and to adjust the training content accordingly if necessary. Finally, sport psychology practitioners are encouraged to promote an athlete’s multidimensional sense of self and a sustainable narrative that continues despite fluctuations in form, performance and potential injuries [64].

### Strengths and Limitations

One of the strengths of the current review is that the literature search was performed by experienced librarians using a thorough strategy. In addition, the risk of bias in the included studies was carefully assessed using validated tools that were modified to account for the objective of this review (i.e. items specific to overuse injury measurement were added). One potential limitation is the relatively small number of included studies. Additional limitations are the heterogeneity in terms of study designs and methods used to measure psychosocial factors and overuse injury, which made the overall certainty of evidence for each factor difficult to appraise. Additionally, a majority of the studies had an unclear or high risk of bias on at least one item. One of the main reasons for the high scores in potential for bias was the use of “intra-personal” means (i.e. self-reported perceptions of the factors) when assessing the “inter-personal factors” and “sociocultural factors”. These issues are especially important to consider when interpreting the findings of this review. Lastly, this review focused on competitive athletes. Therefore, the results cannot be generalised to other populations such as recreational athletes or dancers without caution.

### Future Research Directions

From a methodological standpoint, future research is recommended to more strictly adhere to the recommendations that have been formulated regarding overuse injury definition and recording methods [65]. Regarding the gradual pattern of onset of overuse injuries, intensive repeated-measure designs with, for instance, weekly measurements of psychosocial stress and other potential risk factors, may allow the identification of their relationships with overuse symptoms over time [59, 66]. It is also important that future research investigates the complex processes involving the different psychosocial factors suggested in this review and their potential interactions with physiological mechanisms (e.g. repetitive overload) [51] that may better predict the risk of overuse injury. In this regard, non-linear and complex system paradigms should be considered [52, 67]. In addition, intervention studies aiming at preventing overuse injuries, using psychological techniques, could be designed based on the psychosocial factors identified in this review.

## Conclusions

The findings of this review suggest that psychosocial factors are likely to influence the risk of overuse injuries in competitive athletes. Importantly, these factors and the mechanisms through which they may predispose athletes to overuse injuries appeared to be partially different to those extensively described for traumatic injuries [25–27]. When aiming to reduce the risk of overuse injuries from a psychosocial standpoint, coaches, supporting staff and sport psychologists are therefore encouraged to acknowledge the similarities and differences between traumatic and overuse injury aetiology and to implement preventive measures based on the psychosocial factors identified in this review.

## Supplementary Information

Below is the link to the electronic supplementary material.

Supplementary file1 (PDF 74 kb)

Supplementary file2 (PDF 45 kb)

Supplementary file3 (PDF 89 kb)

Supplementary file4 (PDF 151 kb)

Supplementary file5 (PDF 75 kb)

Supplementary file6 (PDF 295 kb)

## Declarations

### Availability of Data and Material

Evaluation sheets are available upon request.
