# Supplementary material for: Psychosocial Risk Factors for Overuse Injuries in Competitive Athletes: A Mixed-Studies Systematic Review
Source: Sports Med. 2021 Dec 3;52(4):773–88. doi: 10.1007/s40279-021-01597-5 (PMC8938379; doi:10.1007/s40279-021-01597-5)
Supplement: Supplementary file 3 — Supplementary file3 (PDF 89 kb) [file 40279_2021_1597_MOESM3_ESM.pdf]

Documentation of search strategies
University Library search consultation group

Date: March-April 2019

Topic/research question: What are the possible psychological risk factors for overuse injuries in sport?

Name of researcher(s): Ulrika Tranaeus Fitzgerald, IMM

Librarian(s): Carl Gornitzki & Sabina Gillsund

Databases:

1. Medline (Ovid)
2. Web of Science Core Collection
3. Psycinfo (Ovid)

Total number of hits:

- Before deduplication: 6,890
- After deduplication: 5,512

Karolinska Institutet

1. Medline

Interface: Ovid MEDLINE(R) and Epub Ahead of Print, In-Process & Other Non-Indexed Citations and Daily<br><br>Date of Search: 23 April 2019<br><br>Number of hits: 4,136<br><br>Comment: In Ovid, two or more words are automatically searched as phrases; i.e. no quotation marks are needed	Field labels<br><br>• exp/ = exploded MeSH term<br>• / = non exploded MeSH term<br>• .ti,ab,kf. = title, abstract and author keywords<br>• adjx = within x words, regardless of order<br>• * = truncation of word for alternate endings

1	Cumulative Trauma Disorders/	4203
2	Athletic Injuries/	25853
3	(repetiti* adj3 (strain* or injur* or move*)).ti,ab,kf.	3429
4	(strain* adj3 (injur* or syndrom*)).ti,ab,kf.	1821
5	((repetiti* or cumulat*) adj3 (motion* or stress* or trauma*) adj3 (disorder* or injur* or syndrom*)).ti,ab,kf.	788
6	(stress adj3 injur*).ti,ab,kf.	6100
7	(overuse or sport* injur* or athlet* injur*).ti,ab,kf.	14193
8	or/1-7	48881
9	Anxiety/	74309
10	Performance Anxiety/	128
11	exp Stress, Psychological/	121809
12	Resilience, Psychological/	4488
13	Adaptation, Psychological/	89867
14	Life Change Events/	22133
15	Personality/	36599
16	Self-Control/	1718
17	Motivation/	62762
18	Drive/	1951
19	"Aspirations (Psychology)"/	858
20	Achievement/	15072
21	Competitive Behavior/	7307

22	psychology.fs.	984492
23	(adapt* behav* or ambitious* or anguish or anxiety or competi* or coping* or motivat* or passion* or personalit* or person* factor* or psycho* or resilien* or rival* or self blam* or self control* or self regulat* or stressor*).ti,ab,kf.	1222906
24	(risk* adj3 (take* or taking or behav*)).ti,ab,kf.	49042
25	((emotional* or event* or level* or life or perceiv*) adj3 stress*).ti,ab,kf.	56211
26	or/9-25	1997269
27	Athletes/	10759
28	exp Sports/	170799
29	(aikido or archer* or athlete* or athletic* or badminton or ballgame* or ball game* or baseball or basketball or biathl* or bicycle or bicycling or biking or bobsledder* or bobsleigh* or bowling or bowler* or boxing or boxer* or budo or canoe* or climbing or cricket or curling or cycling or decathl* or discus or diver or divers or diving* or dressage* or eventing or equestrian polo or fencer* or fencing or floorball or floor ball or football or golf* or gymnastic* or hammer throw* or handball* or handspring* or handstand* or headstand* or hepathl* or hockey or hurdler* or javelin throw* or judo or jujitsu or karate or kickbox* or kneeboard* or kung fu or lacrosse or luge or marathon* or martial art* or mountaineer* or paddleboard* or parathlet* or para-athlet* or para athlet* or pentathl* or pole vault* or powerlift* or race walk* or rafting* or racquetball or racketball or racket ball or regatta* or rider* or riding or rodeo* or rower* or rowing or rugby or running or runner* or sail* or shot put* or shooter* or shooting* or skateboard* or skater* or skating or skier* or skiing or snowboard* or snowmobil* or soccer or softball or squash or sport* or sprinter* or sprinting or steeplechase* or swim* or tae kwon do or taekwondo or tai or thai or taiji or taijiquan or taichi or "track and field*" or triathl* or trampoline or tumbling or volleyball or water polo or weight* lifting or lifting weight* or wakeboard* or weightlifting or wrestl* or wushu).ti,ab,kf.	329929
30	or/27-29	406045
31	8 and 26 and 30	4179
32	remove duplicates from 31	4176
33	(animals not humans).sh.	4539481
34	32 not 33	4136

2. Web of Science Core Collection

Interface: Clarivate Analytics<br><br>Date of Search: 23 April 2019<br><br>Number of hits: 1,923	Field labels<br><br>• TS/Topic = title, abstract, author keywords and Keywords Plus<br>• NEAR/x = within x words, regardless of order<br>• * = truncation of word for alternate endings

TOPIC: (repetiti* NEAR/3 (strain* OR injur* OR move*)) OR TOPIC: (strain* NEAR/3 (injur* OR syndrom*)) OR TOPIC: ((repetiti* OR cumulat*) NEAR/3 (motion* OR stress* OR trauma*) NEAR/3 (disorder* OR injur* OR syndrom*)) OR TOPIC: (stress NEAR/3 injur*) OR TOPIC: ("overuse" OR "sport* injur*" OR "athlet* injur*") = 33,051

AND

TOPIC: ("adapt* behav*" OR ambitious* OR "anguish" OR "anxiety" OR competi* OR coping* OR motivat* OR passion* OR personalit* OR "person* factor*" OR psycho* OR resilien* OR rival* OR "self blam*" OR "self control*" OR "self regulat*" OR stressor*) OR TOPIC: (risk* NEAR/3 (take* OR "taking" OR behav*)) OR TOPIC: ((emotional* OR event* OR level* OR "life" OR perceiv*) NEAR/3 stress*) = 2,533,459

AND

TOPIC: ("aikido" OR archer* OR athlete* OR athletic* OR "badminton" OR ballgame* OR "ball game*" OR "baseball" OR "basketball" OR biathl* OR "bicycle" OR "bicycling" OR "biking" OR bobsledder* OR bobsleigh* OR "bowling" OR bowler* OR "boxing" OR boxer* OR "budo" OR canoe* OR "climbing" OR "cricket" OR "curling" OR "cycling" OR decathl* OR "discus" OR "diver" OR "divers" OR diving* OR dressage* OR "eventing" OR "equestrian polo" OR fencer* OR "fencing" OR "floorball" OR "floor ball" OR "football" OR golf* OR gymnastic* OR "hammer throw*" OR handball* OR handspring* OR handstand* OR headstand* OR hepathl* OR "hockey" OR hurdler* OR "javelin throw*" OR "judo" OR "jujitsu" OR "karate" OR kickbox* OR kneeboard* OR "kung fu" OR "lacrosse" OR "luge" OR marathon* OR "martial art*" OR mountaineer* OR paddleboard* OR parathlet* OR para-athlet* OR "para athlet*" OR pentathl* OR "pole vault*" OR powerlift* OR "race walk*" OR rafting* OR "racquetball" OR "racketball" OR "racket ball" OR regatta* OR rider* OR "riding" OR rodeo* OR rower* OR "rowing" OR "rugby" OR "running" OR runner* OR sail* OR shot put* OR shooter* OR shooting* OR skateboard* OR skater* OR "skating" OR skier* OR "skiing" OR snowboard* OR snowmobil* OR "soccer" OR "softball" OR "squash" OR sport* OR sprinter* OR "sprinting" OR steeplechase* OR swim* OR "tae kwon do" OR "taekwondo" OR "tai" OR "thai" OR "taiji" OR "taijiquan" OR "taichi" OR "track and field*" OR triathl* OR "trampoline" OR "tumbling" OR "volleyball" OR "water polo" OR "weight* lifting" OR "lifting weight*" OR wakeboard* OR "weightlifting" OR wrestl* OR "wushu") = 753,162

Karolinska Institutet

3. Psycinfo

Interface: Ovid<br><br>Date of Search: 23 April 2019<br><br>Number of hits: 831<br><br>Comment: In Ovid, two or more words are automatically searched as phrases; i.e. no quotation marks are needed<br><br>Note: Set nr 2 including psychological aspects were removed due to the subject coverage in Psycinfo. Thus, only two sets were used in the search strategy.	Field labels<br><br>• exp/ = exploded controlled term<br>• / = non exploded controlled term<br>• .ti,ab,id. = title, abstract and author keywords<br>• adjx = within x words, regardless of order<br>• * = truncation of word for alternate endings

1	(repetiti* adj3 (strain* or injur* or move*)).ti,ab,id.	1176
2	(strain* adj3 (injur* or syndrom*)).ti,ab,id.	185
3	((repetiti* or cumulat*) adj3 (motion* or stress* or trauma*) adj3 (disorder* or injur* or syndrom*)).ti,ab,id.	221
4	(stress adj3 injur*).ti,ab,id.	835
5	(overuse or sport* injur* or athlet* injur*).ti,ab,id.	2623
6	or/1-5	4795
7	exp Athletes/	14135
8	exp Sports/	24766
9	Sport Psychology/	3831
10	Athletic Training/	1553
11	Athletic Performance/	5058
12	Athletic Training/	1553
13	(aikido or archer* or athlete* or athletic* or badminton or ballgame* or ball game* or baseball or basketball or biathl* or bicycle or bicycling or biking or bobsledder* or bobsleigh* or bowling or bowler* or boxing or boxer* or budo or canoe* or climbing or cricket or curling or cycling or decathl* or discus or diver or divers or diving* or dressage* or eventing or equestrian polo or fencer* or fencing or floorball or floor ball or football or golf* or gymnastic* or hammer throw* or handball* or handspring* or handstand* or headstand* or hepathl* or hockey or hurdler* or javelin throw* or judo or jujitsu or karate or kickbox* or kneeboard* or kung fu or lacrosse or luge or marathon* or martial art* or mountaineer* or paddleboard* or	93757

	parathlet* or para-athlet* or para athlet* or pentathl* or pole vault* or powerlift* or race walk* or rafting* or racquetball or racketball or racket ball or regatta* or rider* or riding or rodeo* or rower* or rowing or rugby or running or runner* or sail* or shot put* or shooter* or shooting* or skateboard* or skater* or skating or skier* or skiing or snowboard* or snowmobil* or soccer or softball or squash or sport* or sprinter* or sprinting or steeplechase* or swim* or tae kwon do or taekwondo or tai or thai or taiji or taijiquan or taichi or "track and field*" or triathl* or trampoline or tumbling or volleyball or water polo or weight* lifting or lifting weight* or wakeboard* or weightlifting or wrestl* or wushu).ti,ab,id.	
14	or/7-13	95054
15	6 and 14	831
