# Supplementary material for: Psychosocial Risk Factors for Overuse Injuries in Competitive Athletes: A Mixed-Studies Systematic Review
Source: Sports Med. 2021 Dec 3;52(4):773–88. doi: 10.1007/s40279-021-01597-5 (PMC8938379; doi:10.1007/s40279-021-01597-5)
Supplement: Supplementary file 4 — Supplementary file4 (PDF 151 kb) [file 40279_2021_1597_MOESM4_ESM.pdf]

Supplementary Appendix 3A. Modified Risk of Bias Assessment tool for Non-randomized Studies (RoBANS)

Article: **Decision:**

**SECTION 1: participant sampling**	
**1. The selection of participants**	
Criteria for judgments of a 'Low risk' of bias	**Cohort study**<br>○ Intervention (exposure) and control groups are the same population group (identical institution and period), and the absence of outcomes among the study participants was confirmed at the starting point of the study.<br>○ Inclusion and exclusion criteria are adequately described
	**Case-control study**<br>○ The case and control groups were selected from comparable population groups.<br>○ The case group was clearly defined, and it was clearly demonstrated that the control group is not the patient group.<br>○ Inclusion and exclusion criteria are adequately described.
Criteria for judgments of a 'High risk' of bias	Any one of the following conditions:
	**Cohort study**<br>○ The intervention (exposure) and control groups were selected from different population groups (e.g., the intervention group differs from the control group with respect to study period or study center, or historical control groups were used).<br>○ The presence of outcomes among the study participants was not confirmed at the starting point of the study.
	**Case-control study**<br>○The case and control groups are not the comparable population groups.<br>○ The patient definitions were generated by self-reported or merged data.<br>○ It was not clearly confirmed that the control group excluded patients
Criteria for judgments of an 'Unclear risk' of bias	It is uncertain whether the selection of participants resulted in a „high risk'<br>or a „low risk' of bias
**SECTION 2: measurements used**	
**2. Confounding variables**	
Criteria for judgments of a 'Low risk' of bias	Any one of the following conditions:
	**Non-randomized studies (cohort, case-control or cross-sectional studies)**<br>○ The major confounding variables were adequately confirmed and considered during the design phase

	(e.g., through matching, participation restriction, or other methods).<br>○ The major confounding variables were adequately confirmed and adjusted for during the analysis phase (e.g., through stratification, propensity score approaches, statistical adjustments, or other methods).
Criteria for judgments of a 'High risk' of bias	Any one of the following conditions:
	**Non-randomized studies (cohort, case-control or cross-sectional studies)**<br>○ The major confounding variables were not considered.<br>○ Although the existence of major confounding variables was confirmed, these variables were not adequately considered during the design and analysis phases.
Criteria for judgments of an 'Unclear risk' of bias	It is uncertain whether the confounding variables resulted in a 'high risk' or a „low risk' of bias
**3. Measurement of psychological factors**	
Criteria for judgments of a 'Low risk' of bias	**Non-randomized studies (cohort, case-control or cross-sectional studies)**
	○ Method of psychological factor measurement is adequately valid and reliable to limit misclassification bias (e.g. validated questionnaires or structured interviews)<br>○ A clear definition or description of the factor being assessed is provided<br>○ The psychological factors measured are blinded for outcome measure<br>○ Continuous variables are reported or appropriate cut-offs are used<br>○ The method and setting of measurement are the same for all study participants
Criteria for judgments of a 'High risk' of bias	Any one of the following conditions:
	○ Data were obtained through non-validated tools<br>○ A clear case of interviewer bias*<br>○ A clear case of recall bias**
Criteria for judgments of an 'Unclear risk' of bias	It is uncertain whether the exposure measurement resulted in a „high risk' or a „low risk' of bias
	* "Interviewer bias" describes a situation in which the characteristics of the investigators cause the study data to be standardized in a manner that affects the study results. This phenomenon can be reduced through the training of investigators.

	** “Recall bias” describes a situation in which the respondents' degree of recall can affect the study results.
**4. Attrition biases**	
Criteria for judgments of a 'Low risk' of bias	Any one of the following conditions:
	**Non-randomized studies (cohort, case-control or cross-sectional studies)**<br>○ There are no missing data.<br>○ The causes of any missing data are considered to be relevant to the study outcomes (i.e., censoring does not create a bias in the survival data)<br>○ The quantity of missing data was a product of similar developments in both the intervention (exposure) and the control groups, and the causes of these developments are similar.<br>○ Response rate is adequate and is > 80%<br>○ Reasons for loss to follow up are described
Criteria for judgments of a 'High risk' of bias	Any one of the following conditions:
	**Non-randomized studies (cohort, case-control or cross-sectional studies)**<br>○ The missing data could affect the study outcome. These effects may be attributed to the differences in the missing data between the intervention (exposure) group and the control group, or the effects may be caused by the absence of important measurements.
Criteria for judgments of an 'Unclear risk' of bias	It is uncertain whether the incomplete outcome data resulted in a „high risk' or a „low risk' of bias
**5. Outcome (overuse injury) measurements.**	
Criteria for judgments of a 'Low risk' of bias	Any one of the following conditions:
	**Non-randomized studies (cohort, case-control or cross-sectional studies)**<br>○ The experimental protocol is available, and the pre-defined primary/secondary outcomes were described as planned.<br>○ All of the expected outcomes were included in the study descriptions (even in the absence of the experimental protocols).<br>○ A clear definition of the outcome (overuse as the mechanism of injury) is provided.<br>○ Appropriate methods were used to separate overuse from acute injuries<br>○ The method of outcome measurement is valid and specific to overuse injuries, preferably self-reported measures<br>○ Criteria for the presence of an overuse injury are not only based on a time-loss definition

Criteria for judgments of a 'High risk' of bias	Any one of the following conditions:
	**Non-randomized studies (cohort, case-control or cross-sectional studies)**<br>○ The pre-defined primary outcomes were not fully reported.<br>○ The outcomes were not reported in accordance with the previously defined standards.<br>○ Primary outcomes that were not pre-specified in the study existed (except for outcomes with clear explanations, such as unexpected adverse effects).<br>○ The existence of incomplete reporting regarding the primary outcome of interest.<br>○ The absence of reports on important outcomes that would be expected to be reported for studies in related fields.<br>○ The method and setting of outcome measurement is the same for all study participants
Criteria for judgments of an 'Unclear risk' of bias	It is uncertain whether the selective outcome reporting resulted in a „high risk' or a „low risk' of bias.*
	*Most of the examined studies were classified into this category.
**SECTION 3: analysis**	
**6. Statistical Analysis and Reporting**	
Criteria for judgments of a 'Low risk' of bias	Any one of the following conditions:
	**Non-randomized studies (cohort, case-control or cross-sectional studies)**<br>○ Adequate and specific methods were used to report occurrence/prevalence of overuse injuries (e.g. frequency of symptoms preferred over binary classification)<br>○ A multivariate analysis was undergone to account for interaction between different psychological factors<br>○ There is sufficient presentation of data to assess the adequacy of the analysis<br>○ The strategy for model building is appropriate and is based on a conceptual framework or model.<br>○ There is a description of the association of the prognostic factor and the outcome, including information about the statistical significance and effect sizes<br>○ Continuous variables are reported or cut-off points are used<br>○ There is no selective reporting of results
Criteria for judgments of a 'High risk' of bias	Any one of the following conditions:
	The criteria for a 'low risk of bias are not met

Criteria for judgments of an 'Unclear risk' of bias	It is uncertain whether the selective outcome reporting resulted in a „high risk' or a „low risk' of bias.*
