# Supplementary material for: Psychosocial Risk Factors for Overuse Injuries in Competitive Athletes: A Mixed-Studies Systematic Review
Source: Sports Med. 2021 Dec 3;52(4):773–88. doi: 10.1007/s40279-021-01597-5 (PMC8938379; doi:10.1007/s40279-021-01597-5)
Supplement: Supplementary file 5 — Supplementary file5 (PDF 75 kb) [file 40279_2021_1597_MOESM5_ESM.pdf]

**Supplementary Appendix 3B.** Modified Quality Assessment Scale for Qualitative Studies.

SWEDISH AGENCY FOR HEALTH TECHNOLOGY ASSESSMENT AND ASSESSMENT OF SOCIAL SERVICES

Authors: Year: Article #:

Include in the review: Yes No Borderline

1. Purpose

| | Yes | No | Unclear | Not applicable |
|---|---|---|---|---|
| a) Is the aim clear and defined? | | | | |

2. Selection

| | Yes | No | Unclear | Not applicable |
|---|---|---|---|---|
| a) Is the selection relevant? | | | | |
| b) Is the process of the selection described? | | | | |
| c) Is the context well described? | | | | |
| d) Is the relation researcher/selection sound described? | | | | |
| e) Participants were selected based on a clear definition of overuse injury (clear diagnosis of overuse as the mechanism of injury) | | | | |
| f) Participants had a current overuse injury or just recovered | | | | |

Comments:

3. Data collection

| | Yes | No | Unclear | Not applicable |
|---|---|---|---|---|
| a) Is the data collection well described? | | | | |
| b) Is the data collection relevant? | | | | |
| c) Is there saturation? | | | | |
| d) Have the researchers declared their relation and understanding of the data/subject? | | | | |

Comments:

4. Analysis

| | Yes | No | Unclear | Not applicable |
|---|---|---|---|---|
| a) Is the analysis clear and defined? | | | | |
| b) Is the analysis relevant in relation to the aim and method? | | | | |
| c) Have the researchers declared their understanding in relation to the data/analysis? | | | | |

Comment:

5. Results

| | Yes | No | Unclear | Not applicable |
|---|---|---|---|---|
| a) Is the result logical? | | | | |
| b) Is the result understandable? | | | | |
| c) Is the result sound described? | | | | |
| d) Is the result presented in relation to any theoretical framework? | | | | |

| | | | | | |
|---|---|---|---|---|---|
| e) | Do the result generate future hypothesis/theory/model? | | | | |
| f) | Is the result transferable to similar contexts/populations? | | | | |

Comments:
