# Supplementary material for: Psychosocial Risk Factors for Overuse Injuries in Competitive Athletes: A Mixed-Studies Systematic Review
Source: Sports Med. 2021 Dec 3;52(4):773–88. doi: 10.1007/s40279-021-01597-5 (PMC8938379; doi:10.1007/s40279-021-01597-5)
Supplement: Supplementary file 6 — Supplementary file6 (PDF 295 kb) [file 40279_2021_1597_MOESM6_ESM.pdf]

Supplementary Appendix 4. Full references and reasons for exclusion

References	Eligibility criteria				Decision
	**Empirical study investigating the relationship between at least one psychological variable (as a risk factor) and overuse injuries (as an outcome)**	**Clear definition and measurement of overuse injuries, clear differentiation with acute injuries**	**Population of competitive athletes**	**Methodological quality**	
Bond JW, Miller BP, Chrisfield PM. Psychological prediction of injury in elite swimmers. *Int J Sports Med 1988;9:*345-348.	√	X	√	√	Exclude
Berengui R, Ortin FJ, de Los Fayos EJG, et al. Personlity and injuries in high performance sports. *Revista Iberoamericana De Psicologia Del Ejercicio Y El Deporte* 2017;12:15-22.	√	√	√	√	Include
Brewer BW. Causal attribution dimensions and adjustment to sport injury. *Journal of Personal & Interpersonal Loss 1999;4:*215-224.	X	√	√	√	Exclude
Cavallerio F, Wadey R, Wagstaff CRD. Understanding overuse injuries in rhythmic gymnastics: A 12-month ethnographic study. *Psychol Sport Exerc* 2016;25:100-09.	√	√	√	√	Include
Christensen D, Ogles B. Injury description and prediction in marathon runners. *Int J Sport Psychol* 017;48:660-74.	√	√	√	√	Include
Ekenman I, Hassmen P, Koivula N, et al. Stress fractures of the tibia: can personality traits help us detect the injury-prone athlete? *Scand J Med Sci Sports* 2001;11:87-95.	√	√	√	√	Include

Galambos SA, Terry, PC, Moyle GM, Locke SA. Psychological predictors of injury among elite athletes. *Br J Sports Med 2005;39:*351-354.	X	√	√	√	Exclude
Habif SE. *Examination of injury and the association between sport injury anxiety and injury severity and frequency among Olympic distance triathletes* 2008 (Doctoral dissertation, Teachers College, Columbia University).	√	X	√	√	Exclude
Heidari J, Mierswa T, Kleinert J, et al. Parameters of low back pain chronicity among athletes: associations with physical and mental stress. *Physical Therapy in Sport 2016;21:*31-37.	X	√	√	√	Exclude
Heidari J, Hasenbring M, Kleinert J, et al. Stress-related psychological factors for back pain among athletes: Important topic with scarce evidence. *Eur J Sport Sci* 2017;17:351-359.	X	√	√	√	Exclude
Jelvegard S, Timpka T, Bargoria V, et al. Perception of Health Problems Among Competitive Runners A Qualitative Study of Cognitive Appraisals and Behavioral Responses. *Orthopaedic Journal of Sports Medicine* 2016;4	√	√	√	√	Include
Laux P, Krumm B, Diers M, et al. Recovery–stress balance and injury risk in professional football players: a prospective study. *J Sports Sci* 2015;33:2140-2148.	√	X	√	√	Exclude
Lysens RJ, Ostyn M S, Auweele YV, et al. The accident-prone and overuse-prone profiles of the young athlete. *The American Journal of Sports Medicine*, 1989;*17:* 612-619.	√	√	X	√	Exclude
Madigan DJ, Stoeber J, Forsdyke D, et al. Perfectionism predicts injury in junior athletes:	√	X	√	√	Exclude

Preliminary evidence from a prospective study. *J Sports Sci 2018;36:*545-550.					
Martin S, Johnson U, McCall A, Ivarsson A. Psychological risk profile for overuse injuries in sport: an exploratory study (forthcoming). Forthcoming.	√	√	√	√	Include
Pensgaard AM, Ivarsson A, Nilstad A, et al. Psychosocial stress factors, including the relationship with the coach, and their influence on acute and overuse injury risk in elite female football players. *BMJ open sport & exercise medicine* 2018;4(1):e000317.	√	√	√	√	Include
Russell HC, Wiese-Bjornstal DM. Narratives of Psychosocial Response to Microtrauma Injury among Long-Distance Runners. *Sports* 2015;3:159-77.	√	√	√	√	Include
Steffen K, Pensgaard, AM, Bahr R. Self-reported psychological characteristics as risk factors for injuries in female youth football. *Scand J Med Sci Sports 2009;19:*442-451.	√	X	√	√	Exclude
Timpka T, Jacobsson J, Dahlstrm O, et al. The psychological factor 'self-blame' predicts overuse injury among top-level Swedish track and field athletes: a 12-month cohort study. *Br J Sport Med* 2015;49(22)	√	√	√	√	Include
Tranaeus U, Johnson U, Engstrom B, et al. Psychological antecedents of overuse injuries in Swedish elite floorball players. *Athletic Insight* 2014;6:155.	√	√	√	√	Include
van der Does HTD, Brink MS, Otter RTA, et al. Injury Risk Is Increased by Changes in Perceived	√	√	√	√	Include

Recovery of Team Sport Players. *Clin J Sport Med* 2017;27:46-51.					
Van der Sluis A, Brink MS, Pluim B, et al. Is risk-taking in talented junior tennis players related to overuse injuries? *Scand J Med Sci Sports* 2017;27:1347-55.	√	√	√	√	Include
van der Sluis A, Brink MS, Pluim BM, et al. Self-regulatory skills: Are they helpful in the prevention of overuse injuries in talented tennis players? *Scand J Med Sci Sports* 2019;29:1050-58.	√	√	√	√	Include
van Wilgen CP, Verhagen E. A qualitative study on overuse injuries: The beliefs of athletes and coaches. *J Sci Med Sport* 2012;15:116-21.	√	√	√	√	Include
Vetter RE, Symonds ML. Correlations between injury, training intensity, and physical and mental exhaustion among college athletes. *The Journal of Strength & Conditioning Research 2010;24:*587-596.	√	√	√	X	Exclude
Wickström W, Spreco A, Bargoria V, et al. Perceptions of overuse injury among Swedish ultramarathon and marathon runners: cross-sectional study based on the Illness Perception Questionnaire Revised (IPQ-R). *Frontiers in psychology 2019;10:*2406.	√	X	√	√	Exclude
Wippert PM, Puschmann AK, Arampatzis A, et al. Diagnosis of psychosocial risk factors in prevention of low back pain in athletes (MiSpEx). *BMJ open sport & exercise medicine 2017;3:* e000295.	X	√	√	√	Exclude

√ criterion met; X criterion unmet.
